# Two new species of Microcotylidae Taschenberg, 1879 (Platyhelminthes: Polyopisthocotyla) parasitising *Diplodus capensis* (Teleostei, Sparidae) off South Africa

**DOI:** 10.1051/parasite/2025037

**Published:** 2025-07-25

**Authors:** Anja Vermaak, Chahinez Bouguerche, Aline A. Acosta, Nico J. Smit

**Affiliations:** 1 Water Research Group, Unit for Environmental Sciences and Management, North-West University Potchefstroom 2531 South Africa; 2 Department of Zoology, Swedish Museum of Natural History Box 50007 Stockholm SE-104 05 Sweden; 3 Department of Biological Sciences, Klein College of Science, University of North Carolina at Charlotte Charlotte North Carolina 28223-0001 USA

**Keywords:** Microcotylidae, *Atriaster*, *Polylabris*, Marine fish parasites, Integrative taxonomy, Life under water

## Abstract

Microcotylids have rarely been reported along the South African coast, even though the Microcotylidae is one of the dominant polyopisthocotylan families. The present study focused on elucidating the parasite diversity of the Cape white seabream, *Diplodus capensis* (Smith), from various localities along the South African coast. By combining molecular and morphological techniques, two previously undescribed species of the Microcotylidae were identified. *Atriaster ibamba* n. sp. primarily differs from its congeners by the number and size of the hooks surrounding the genital atrium. *Polylabris dassie* n. sp. has a single vagina and is unique to most others of this genus by having a smaller male copulatory organ, and by the shape of this organ. This is the first report of species of *Atriaster* from South Africa, as well as the first report of any polyopisthocotylan from *D*. *capensis*. The present study also contributes the first genetic sequences of marine microcotylids from South Africa.

## Abstract (fr)

Les Microcotylidae ont rarement été signalés le long des côtes sud-africaines, bien cette famille soit l’une des plus nombreuses des Polyopisthocotyla. La présente étude s’est attachée à élucider la diversité parasitaire du sar commun du Cap, *Diplodus capensis* (Smith) dans diverses localités de la côte sud-africaine. En combinant des techniques moléculaires et morphologiques, deux espèces de Microcotylidae jusque-là non décrites ont été identifiées. *Atriaster ibamba* n. sp. se distingue principalement de ses congénères par le nombre et la taille des crochets entourant l’atrium génital. *Polylabris dassie* n. sp. possède un seul vagin et se distingue de la plupart des autres espèces de ce genre par son organe copulateur mâle plus petit et par la forme de cet organe. Il s’agit de la première mention d’une espèce d’*Atriaster* en Afrique du Sud, ainsi que de la première mention d’un Polyopisthocotyla chez *D. capensis*. Cette étude fournit également les premières séquences génétiques de Microcotylidae marins d’Afrique du Sud.

## Introduction

The diversity of polyopisthocotylans of the Microcotylidae Taschenberg 1879 hosted by marine fishes along the South African coast remains largely uncharted. To date, only a single microcotylid species, *Polylabris madagascarensis* Hayward, 1996 from the silver sillago, *Sillago sihama* (Fabricius), has been reported from this region [26]. This is noteworthy considering that the Microcotylidae is one of the largest polyopisthocotylan families, consisting of about seven subfamilies and 51 genera [4]. Given the very high biodiversity supported by the unique marine habitats along the South African coast, one would anticipate greater diversity of these parasites [62].

The Cape white seabream, *Diplodus capensis* (Smith) (Teleostei, Sparidae), is commonly found in a variety of near-shore habitats spanning the southern African coast from Angola to Mozambique [27]. It is popular among shore anglers and is integral in subsistence fishing, particularly in regions like southern Angola [58]. Therefore, this fish species holds both significant ecological and socioeconomic importance. Thus far, only five metazoan parasites have been described or reported from this fish host: the digeneans *Holorchis pycnoporus* Stossich, 1901 [9] and *Proctoeces maculatus* (Looss, 1901) [67]; the copepod *Caligus epinepheli* Yamaguti, 1936 [22]; as well as the isopods *Ceratothoa famosa* Hadfield, Bruce & Smit, 2014 [25], and *Gnathia pilosus* Hadfield, Smit & Avenant-Oldewage, 2008 [24]. Notably, no descriptions or reports of polyopisthocotylan species have been published from this fish host thus far.

During an in-depth study aimed at unravelling the diversity of metazoan parasites from *D*. *capensis*, two species of microcotylids (Prostatomicrocotylinae Yamaguti, 1968 and Atriasterinae Maillard & Noisy, 1979) were collected. A detailed morphological and molecular study revealed that the microcotylids obtained from the gills of *D*. *capensis* from South Africa represent species new to science.

## Material and methods

### Host and parasite collection and ethics

**Figure 1:**
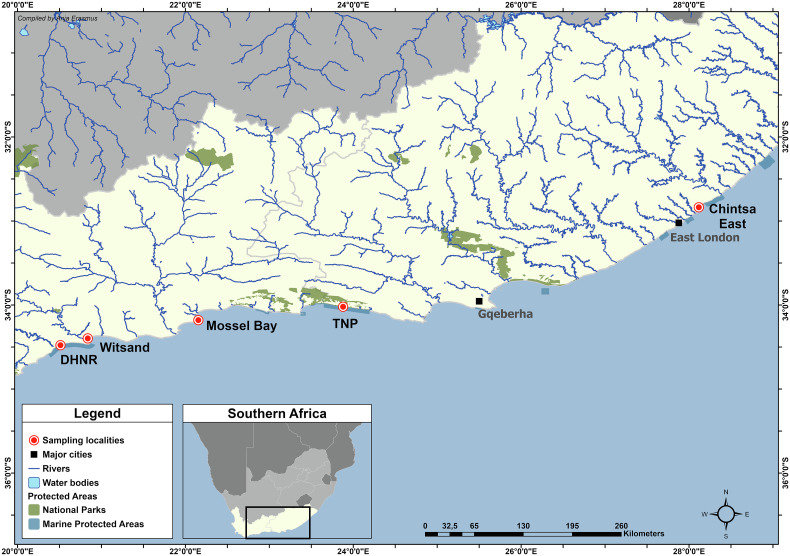
Map of sampling localities along the South African coast. Abbreviations: DHNR, De Hoop Nature Reserve; TNP, the Tsitsikamma section of the Garden Route National Park.

Sixty-eight specimens of *D. capensis* were collected using rod and reel from five localities along the South African coast: Koppie Alleen, De Hoop Nature Reserve (DHNR) (*n* = 12), Witsand (*n* = 3), Mossel Bay (*n* = 5), the Tsitsikamma section of the Garden Route National Park (TNP) (*n* = 29), and Chintsa East (*n* = 19) (Fig. 1). After being humanely killed by a combination of stunning, cranial pithing and spinal severance, the fish were subjected to full parasite screening. Sampling was conducted under the following permits: CN44-87-18289 for De Hoop Nature Reserve; RES2020/29, RES2021/49, and RES2022/44 for Witsand, Mossel Bay, and Chintsa; and MALH-K2016-005a and SMIT-NJ/2020-004 for the Tsitsikamma section of the Garden Route National Park. Ethics clearance for this research was received from the North-West University AnimCare ethics committee (NWU-00759-22-A5). Fish nomenclature and common names followed FishBase [21]. Calculations of prevalence and intensity of infection followed Bush *et al.* [10].

### Morphological methods

Polyopisthocotylans were heat-fixed without pressure in near-boiling saline and preserved immediately in 80% ethanol for parallel morphological and molecular characterisation. A total of 13 specimens were processed as hologenophores (sensu Pleijel *et al.* [55]). A small lateral part of the polyopisthocotylan was excised with a scalpel blade and used for molecular analyses, whereas the rest of the specimen was stained and mounted on a slide. Whole-mounts and hologenophores for morphological analyses were stained with acetic carmine, destained with diluted hydrochloric acid, dehydrated in an ethanol series (70%, 96% and 100%), cleared in clove oil and mounted in Canada balsam. Drawings were made with the aid of a Nikon Eclipse *Ni*-U Microscope (Nikon Instruments, Tokyo, Japan) with DIC (differential interference contrast) and a drawing tube. Drawings were scanned and digitised using Adobe Illustrator 2023. Measurements of whole-mounts and hologenophores are in micrometres (μm) and given as a range followed by the mean in parentheses. Measurements of newly characterised species and other valid species of *Atriaster* and *Polylabris* are given in the Supplementary material (Table S1).

For scanning electron microscopy, specimens were dehydrated in a graded ethanol series, followed by a graded hexamethyldisilazane (HMDS) series. Thereafter, specimens were sputter-coated with a mixture of gold and palladium and photographed using a Phenom Pro Desktop scanning electron microscope (Thermo Scientific, Waltham, MA, USA).

### Nomenclature

Designation of the ventral and dorsal arms of clamp sclerites followed Bouguerche *et al.* [6]. Nomenclature for the male terminal genitalia of *Atriaster* followed Mamaev and Parukhin [45], and we considered that the male copulatory apparatus consists of 3 types of hooks: 1. genital atrium hooks: hooks on the walls of the genital atrium forming a crown; 2. paired hooks: a pair of long hooks on the muscular thickening on the anterior part of the atrium; 3. copulatory organ hooks: hooks on the muscular pad in the centre of the genital atrium. For high-level terminology of the Polyopisthocotyla, we followed the systematics of Brabec *et al.* [7] who recently elevated the former subclasses of “Monogenea” to the level of classes. Voucher material and type specimens were deposited in the parasite collection of the National Museum in Bloemfontein (NMB), South Africa; and the Swedish Museum of Natural History (SMNH) in Stockholm, Sweden.

### Molecular methods

#### Generation of molecular data

DNA was extracted from the excised pieces of the worms using the PCRBiosystems Rapid DNA Extraction Kit (PCRBiosystems available from Analytical Solutions, Randburg, South Africa). The manufacturer’s protocol was followed, except only 100 μL were added during the final dilution step, instead of the recommended 900 μL, to obtain a higher concentration of DNA. The partial 28S nuclear ribosomal RNA gene and the mitochondrial cytochrome *c* oxidase subunit I (COI) gene were amplified by polymerase chain reaction (PCR). The 28S gene was amplified using the forward primer U178 (5′–GCA CCC GCT GAA YTT AAG–3′) and reverse primer L1642 (5′–CCA GCG CCA TCC ATT TTCA–3′) [38], following the protocol of Acosta and Smit [1]. The primer set of Asmit1 (5′–TTT TTT GGG CAT CCT GAG GTT TAT–3′) and Asmit2 (5′–TAA AGA AAG AAC ATA ATG AAA ATG–3′) [36] were used to amplify the COI gene of the *Polylabris* species, utilising the following protocol: denaturation at 95 °C for 15 min; followed by 35 cycles of 94 °C for 1 min, 48 °C for 2 min and 72 °C for 2 min; and a final extension of 72 °C for 10 min. Unfortunately, it was not possible to amplify the COI gene of the *Atriaster* species, after trials with various primer combinations and PCR conditions. PCR amplicons were visualised with 1% gel electrophoresis and sent to a commercial sequencing company (Inqaba Biotechnical Industries (Pty). Ltd., Pretoria, South Africa) for purification and sequencing. The new sequences were assembled with Geneious v. 11.1.4 bioinformatics software (Biomatters, Auckland, New Zealand). GenBank accession numbers are included in Table 1.

**Table 1:** Sequences used in the phylogenetic analyses.

Species	Host	Locality	GenBank accession numbers		Reference
			28S	COI	
***Atriaster ibamba* n. sp.**	*Diplodus capensis* (Smith)	Witsand, SA	PV658388	–	Present study
		Mossel Bay, SA	PV658387	–	Present study
		DHNR, SA	PV658386	–	Present study
			PV658383		
		TNP, SA	PV658384	–	Present study
			PV658385		
			PV658389		
*Atrispinum acarne* Maillard & Noisy, 1979	*Pagellus acarne* (Risso)	Sète, France	AF311702	–	[30]
	*Diplodus vulgaris* (Geoffroy Saint-Hilaire)	Algeria	OL679672	OL675203-05	[32]
*Bychowskicotyla mormyri* (Lorenz, 1878) Mamaev, 1984	*Lithognathus mormyrus* (L.)	France	AF311713 ^a^	–	[30]
*Bivagina pagrosomi* (Murray, 1931)	Unspecified	Unspecified	AJ243678	–	[37]
*Lutianicola* sp.	Unspecified	Unspecified	MH700259	–	Unpublished
*Microcotyle arripis* Sandars, 1945	*Arripis georgianus* (Valenciennes)	Off South Australia	GU263830	–	[13]
*Microcotyle erythrini* Van Beneden & Hesse, 1863	*Pagellus erythrinus* (L.)	France	AM157221	–	[2]
	*Pagrus pagrus* (L.)	Western Mediterranean	MN814849	–	[69]
	*P*. *pagrus*	Algeria	OL679676	–	[32]
*Microcotyle isyebi* Bouguerche, Gey, Justine & Tazerouti, 2019	*Boops boops* (L.)	Western Mediterranean	MN814850	–	[69]
*Microcotyle sebastis* Goto, 1894	*Sebastes* sp.	North Sea, UK	AF382051	–	[53]
*Microcotyle whittingtoni* Víllora-Montero, Pérez-del-Olmo, Georgieva, Raga & Montero, 2020	*Dentex dentex* (L.)	Western Mediterranean	MN814847	–	[69]
*Microcotyloides incisus* (Linton, 1910)	*Lutjanus griseus* (L.)	Mexico	MG586861	–	[48]
*Omanicotyle heterospina* (Mamaev & Parukhin, 1974)	*Argyrops spinifer* (Forsskål)	Arabian Sea	JN602095 ^b^	–	[77]
*Plectanocotyle gurnardi* (Van Beneden & Hesse, 1863) Llewellyn, 1941	*Eutrigla gurnardus*	Sweden	–	PP297655	[12]
***Polylabris dassie* n. sp.**	*D*. *capensis*	Witsand, SA	PV627795	PV612823	Present study
			PV627796	PV612824	
		Mossel Bay, SA	PV627797	PV612825	Present study
		DHNR, SA	PV627798	PV612826	Present study
		Chintsa East, SA	PV627799	PV612827	Present study
*Polylabris australiensis* Hayward, 1996	Unspecified	Australia	–	MZ273906-08	Unpublished
*Polylabris* cf. *mamaevi*	Unspecified	Unspecified	MH700591	–	Unpublished
*Polylabris halichoeres* Wang & Zhang, 1998	Unspecified	Unspecified	–	JF505509	[80]
				NC016057	
*Polylabris sillaginae* (Woolcock, 1936)	*Sillaginodes punctatus* (Cuvier)	Off South Australia	GU289509	–	[13]
	Unspecified	Australia	–	MZ273898-9	Unpublished
				MZ273900-05	
*Polylabris* sp.	Unspecified	Unspecified	MH700257	–	Unpublished
*Polylabroides* sp.	Unspecified	Unspecified	MH700258	–	Unpublished
Prostatomicrocotylinae gen. sp.	*Diplodus vulgaris* (Geoffroy Saint-Hilaire)	Algeria	–	OL675212	[32]
*Sparicotyle chrysophrii* (Van Beneden & Hesse, 1863)	*Sparus aurata* (L.)	Sète, France	AF311719	AY009161	[30]
	*S. aurata*	Algeria	OL679674	OL675206	[32]
				OL675207	
				OL675208	
				OL675209	
				OL675210	
		Croatia		GQ240236	[51]

Abbreviations: DHNR, De Hoop Nature Reserve; TNP, the Tsitsikamma section of the Garden Route National Park; SA, South Africa. Reported as *Pagellicotyle mormyri* on GenBank;

^a^Reported as *Pagellicotyle mormyri* on GenBank. Newly described species are highlighted in bold.

^b^ Reported on GenBank as Microcotylidae gen. sp.

#### Phylogenetic analyses

Phylogenetic analyses were performed using the newly generated sequences of the microcotylids and those of closely related species available in GenBank (Table 1). Alignments for each gene region were generated using the default parameters of MUSCLE [16] as implemented in Geneious v. 7.1.3. Nucleotide substitution models for phylogenetic analyses were calculated using MEGA 7 [31]. Both 28S and COI alignments were subjected to the model GTR + I + G for phylogenetic analyses. RAxML [23] was used to generate maximum likelihood (ML) trees, estimating model parameters and bootstrap support values (1,000 repetitions). MrBayes [59] was used to generate Bayesian Inference (BI) trees, running two independent MCMC runs of four chains for 10 million generations and sampling tree topologies every 1,000 generations. Burn-in periods were set to the first 25,000 generations. Both ML and BI analyses were carried out on the computational resource CIPRES [50]. FigTree v1.4.4 was used to visualise the phylogenetic trees [57]. Genetic distance matrices were calculated using MEGA 7.

## Results

Two species of microcotylids were found parasitising *D. capensis*. Based on morphological analyses, one species belongs to *Atriaster* and the other to *Polylabris*. Data on the prevalence and intensity of infestation for both species are presented in Table 2. The two species are regarded as new to science and are described below.

**Table 2:** Prevalence and intensity of infection of the two microcotylid species infecting *Diplodus capensis* in South Africa, found in the present study (DHNR, De Hoop Nature Reserve; TNP, Tsitsikamma section of the Garden Route National Park).

Species	Locality	Number of fish collected	Prevalence	Intensity of infection
*Atriaster ibamba* n. sp.	DHNR	12	75%	1–12
	Witsand	3	2 of 3	1–2
	Mossel Bay	5	4 of 5	4–5
	TNP	29	55.2%	1–6
	Chintsa East	19	42.1%	1–5
*Polylabris dassie* n. sp.	DHNR	12	41.7%	1–5
	Witsand	3	1 of 3	0–2
	Mossel Bay	5	2 of 5	0–1
	TNP	29	10.3%	0–1
	Chintsa East	19	26.3%	1–4

### Morphological characterisation

Class Polyopisthocotyla Brabec, Salomaki, Kolísko, Scholz & Kuchta, 2023

Family Microcotylidae Taschenberg, 1879

Subfamily Atriasterinae Maillard & Noisy, 1979

Genus *Atriaster* Lebedev & Parukhin, 1969

### *Atriaster ibamba* n. sp. (Figs. 2–4)

urn:lsid:zoobank.org:act:96DFF280-90ED-4093-BAD5-DD1B0C9A386F

**Figure 2:**
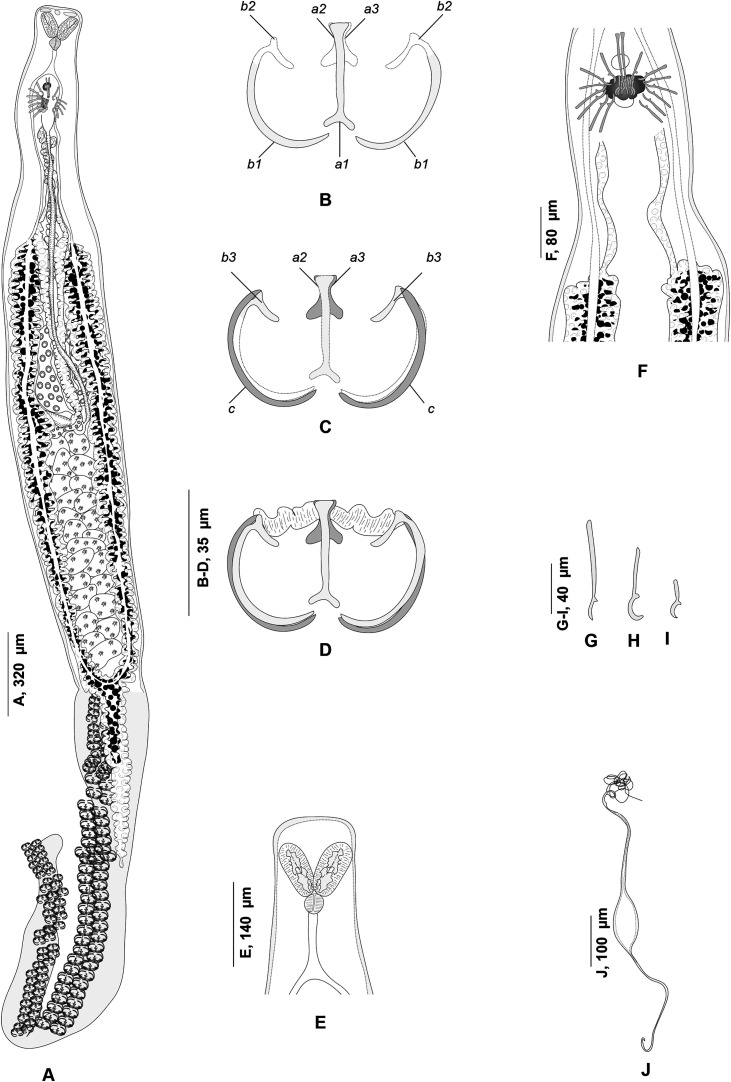
*Atriaster ibamba* n. sp. ex *Diplodus capensis* from South Africa. A, body, ventral view (NMBP1091). B, organisation of clamps sclerites in ventral jaw. C, organisation of clamps sclerites in dorsal jaw. D, clamp, ventral view (NMBP1109). E, anterior end showing rims of oral suckers (NMBP1111). F, anterior part showing male copulatory apparatus (NMBP1091). G, paired central hooks. H, outer hooks. I, inner hooks (NMBP1111). J, egg (NMBP1116).

**Figure 3:**
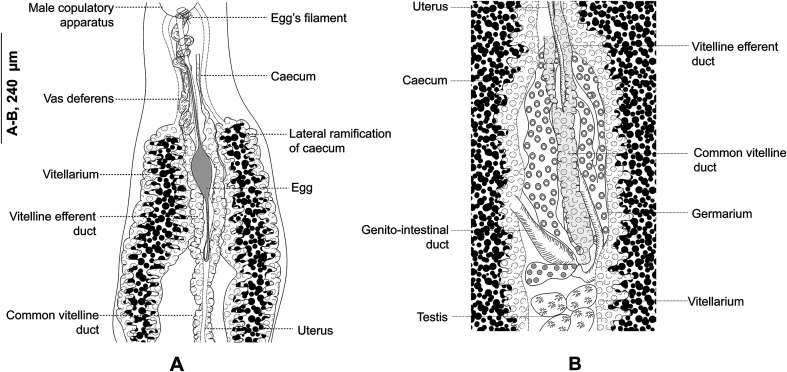
*Atriaster ibamba* n. sp. ex *Diplodus capensis* from South Africa. A, detail of anatomy in anterior body (NMBP1112). B, details of ovarian region (NMBP1114).

**Figure 4:**
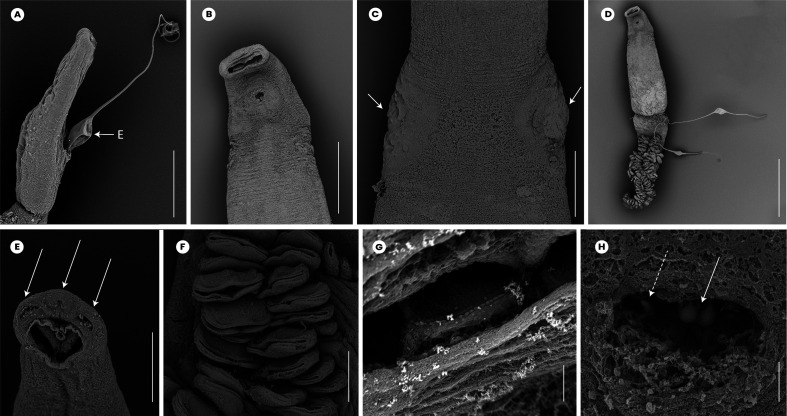
*Atriaster ibamba* n. sp. scanning electron micrographs [SEM]. A, anterior forebody, lateral view, parasite depositing egg; B, anterior end, ventral view; C, anterior end, arrows pointing to lateral vaginal openings; D, full body, ventral view, eggs tangled around posterior end of body; E, anterior extremity, arrows indicating apical glands; F, clamps; G, septate oral suckers; H, genital pore, solid arrow indicating paired median hooks, dotted arrow indicating lateral hooks. Abbreviations: E, egg. Scale bars: A, B, 200 μm; C, 80 μm; D, 500 μm; E, F, 50 μm; G, H, 10 μm.

*Type-host*: *Diplodus capensis* (Smith) (Sparidae), Cape white seabream.

*Type-locality*: Koppie Alleen, De Hoop Nature Reserve, South Africa (34°28′42.1”S, 20°30′39.9”E).

*Other localities*: Witsand (34°23′49″S, 20°50′14.71”E), Mossel Bay (34°10′45.3″S, 22°09′07.4″E), the Tsitsikamma section of the Garden Route National Park (34°1′15.2112″S, 23°52′43.2264″E), and Chintsa East (32°50′11.5368″S, 28°7′1.1892″E), South Africa.

*Site of infestation*: gills.

*Deposited examined material*: Holotype (NMBP1091) and 33 paratypes (NMBP1092–NMBP1124), including four hologenophores, are deposited in the NMB; six paratypes deposited in SMNH (Type-9977–Type-9982).

*Representative DNA sequences*: Partial 28S rDNA, seven sequences (PV658383–PV658389).

*Etymology*: The species epithet ibamba, noun in apposition, is an isiXhosa word meaning “to grip”, “to hold on” or “to clamp on”. This refers to the method of attachment of this group of polyopisthocotylans.

#### Description

Body lanceolate (Fig. 2A), anterior part of body slender, with slight constriction at level of genital atrium in some specimens; body 3443 (1644–5481) long including haptor length, body (excluding haptor) 2264 (1181–3865) long; 371 (204–636) wide at level of testes. Haptor elongate, 1658 (315–2489) long, with 115–228 (168) clamps of “microcotylid” type, sessile, situated in two parallel rows, decreasing in size antero-posteriorly; anterior clamps 35 (22–57) long, 64 (42–109) wide; posterior clamp 29 (20–41) long, 51 (33–80) wide. Clamps formed by anterior (Fig. 2B) and posterior jaws (Fig. 2C). Ventral arm of median spring *a1* Y-shaped, long, slender; distal part of *a1* Y-shaped, with pointed short branches of equal size; proximal part *a2* wider, T-shaped, with short branches. Dorsal arm of median spring *a3* shorter than *a1*, distally broad. Ventral arm of ventral jaw sclerites *b1*, dorsal arm *b2* short and curved inwards, *b2* not reaching median spring. Dorsal jaw sclerites *c* shorter than ventral, sclerites *c* not reaching midline on distal side. Muscle connecting *a2* and *b2* present on proximal side (Fig. 2D).

Prohaptoral suckers transversely oval, 85 (60–111) long, 42 (34–51) wide, muscular, septate, divided by transverse muscular partition into two subequal chambers, lateral chamber smaller; rims of buccal suckers with rows of minute conical expansions (Fig. 2E). Three groups of apical glands, two larger lateral groups and one smaller median group in anterior extremity. Pharynx spherical to subspherical, 36 (27–51) long, 37 (28–44) wide. Oesophagus relatively long. Intestinal bifurcation anterior to genital pore; caeca with numerous short lateral ramifications, uniting posteriorly, forming diverticulum that extends along anterior portion of haptor axis.

Testes 23 (8–35) in number, postovarian, intercaecal, in posterior half of body, distributed in two rows. Copulatory apparatus consisting of three types of hooks: paired central hooks located on the muscular thickening in anterior part of genital atrium; outer hooks located on the walls of the genital atrium forming a crown; and inner hooks located on muscular pad in middle of genital atrium (Fig. 2F). Paired hooks 84 (55–105) long, rod-shaped, visibly curved ventrally (Fig. 2G). Outer hooks 16 (12–21) in number, 57 (30–72) long, thin, with distal curved ends facing interior of genital atrium (Fig. 2H). Inner hooks robust, 8 (6–10) in number, 39 (25–64) long, located in middle of the genital atrium in well mounted specimens (Fig. 2I). Genital atrium with thick muscular walls, opening ventrally as a transversely elongate pore. Vas deferens sinuous, opening into genital atrium anterodorsally (Fig. 3A).

Germarium pretesticular, intercaecal, dorsal to vitelline ducts and uterus, in form of question mark (Fig. 3B). Uterus extending anteriorly to genital atrium. Genito-intestinal duct short, projecting into right caecum. Two lateral vaginae opening between genital pore and anterior extent of vitellarium; vaginae unarmed, tegument covered with numerous small filaments in this area. Vitelline follicles dispersed in two lateral fields surrounding caeca, extending within haptor; anterior limit of vitellarium far posterior to intestinal bifurcation. Vitelline ducts Y-shaped, with two separate efferent ducts, joining in common deferent duct, ventral, at germarium level. Eggs fusiform, with bipolar filaments; anterior filament long and coiled, often coiled around next egg; posterior filament shorter, digitiform (Fig. 2J).

#### Remarks

*Atriaster* currently consists of five nominal species [70]. *Atriaster ibamba* n. sp. fits the diagnosis of the genus well by having a pair of longer median hooks in the genital atrium, two vaginae, and a supporting ribbed plate in the genital atrium [19, 35, 45]. This species differs from *Atriaster acanthopagri* Mamaev & Parukhin, 1975 by having fewer (16 *vs* 40) and longer (30–72 *vs* 41–45) outer hooks, longer (84 *vs* 70) paired hooks, and by having more (8 *vs* 5) and longer (25–64 *vs* 40–45) inner hooks [45]. It differs from *Atriaster bifidacanthus* Mamaev & Parukhin, 1975 by having fewer (12–21 *vs* 35–36) and shorter (30–72 *vs* 70–80) outer hooks, shorter paired hooks (55–105 *vs* 140–160), and by having fewer (6–10 *vs* 12–13) and smaller (25–64 *vs* 70–80) inner hooks [45].

*Atriaster ibamba* n. sp. is distinguished from *Atriaster spinifer* Mamaev & Parukhin, 1975 by having fewer (12–21 *vs* 32–35) and longer (30–72 *vs* 37–45) outer hooks, longer (55–105 *vs* 53–74) paired hooks, and by having more (6–10 *vs* 4–5) and longer (25–64 *vs* 32–40) inner hooks [45]. It differs from *Atriaster maillardi* Lopez-Roman & De Armas Hernandez, 1989 by having a smaller body (315–2489 × 204–636 *vs* 1320–5460 × 391–773), smaller clamps (22–57 × 42–109 *vs* 27–41 × 32–68) and fewer outer hooks (12–21 *vs* 20–35) [39].

One species, *Atriaster heterodus* Lebedev & Parukhin, 1969 was described from a locality off Walvis Bay, Namibia, within the distribution range of the host *D. capensis* [35]. *Atriaster ibamba* n. sp. differs from the former species by having more (115–228 *vs* 120–160) and smaller (35 × 64 *vs* 52 × 90) clamps, shorter outer hooks (30–72 *vs* 70–76), shorter paired hooks (55–105 *vs* 112–118), and by having fewer (6–10 *vs* 8–9) and shorter (25–64 *vs* 70–174) inner hooks.

Subfamily Prostatomicrocotylinae Yamaguti, 1968

Genus *Polylabris* Euzet & Cauwet, 1967

### *Polylabris dassie* n. sp. (Figs. 5 & 6)

urn:lsid:zoobank.org:act:9636659A-D824-4EAE-9CD2-8AB7D33DAC7F

**Figure 5:**
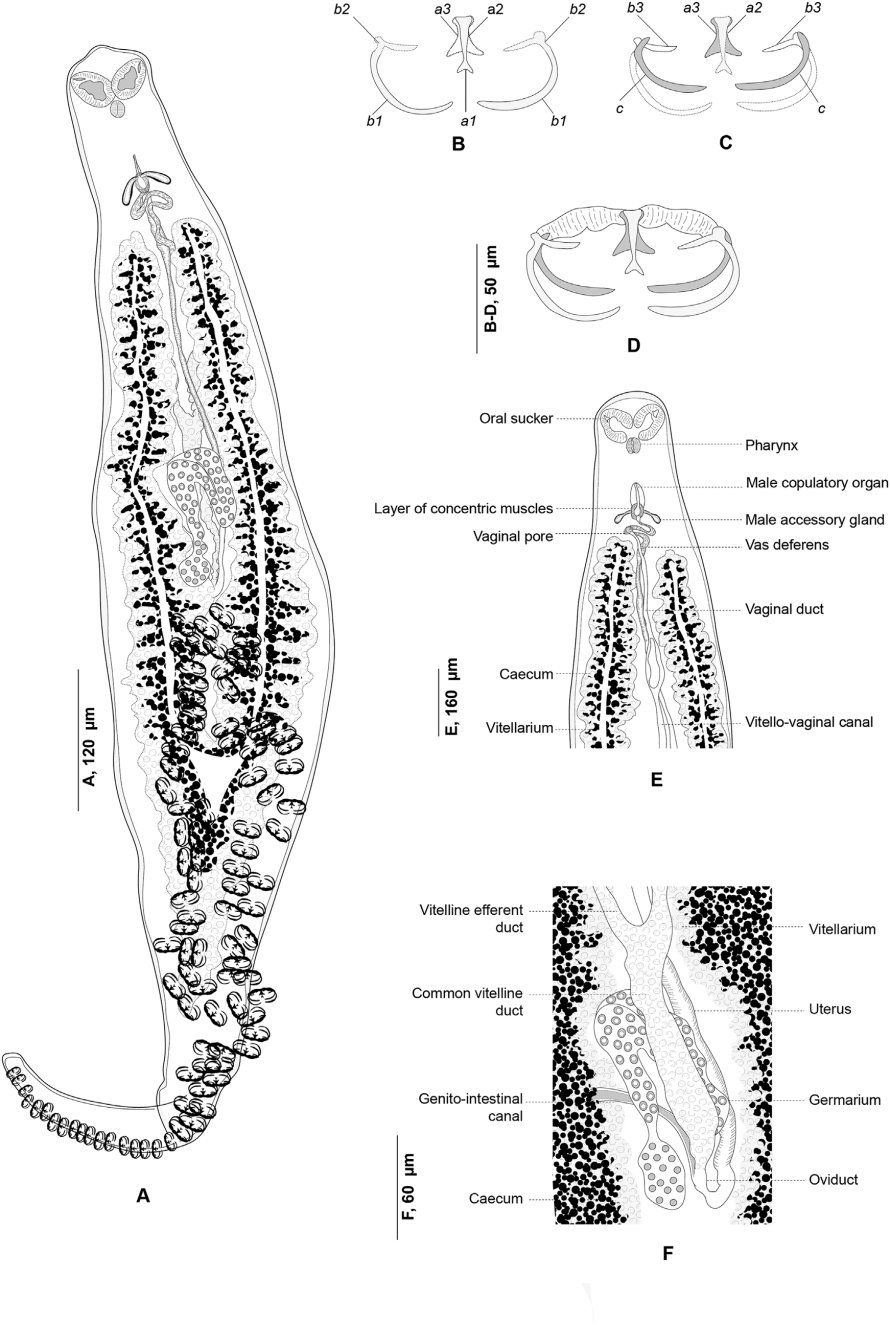
*Polylabris dassie* n. sp. ex *Diplodus capensis* from South Africa. A, body, ventral view (NMBP1125). B, organisation of clamps sclerites in ventral jaw. C, organisation of clamps sclerites in dorsal jaw. D, clamp, ventral view (NMBP1125). E, anterior end showing male copulatory organ (Type-9987). F, detail of the ovarian region.

**Figure 6:**
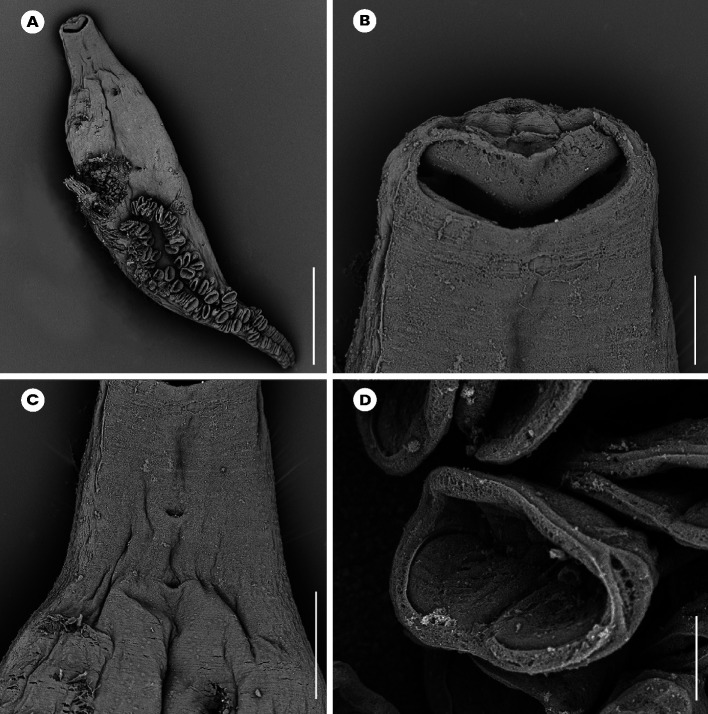
*Polylabris dassie* n sp. scanning electron micrographs [SEM]. A, whole body, ventral view; B, anterior extremity with oral suckers; C, genital openings; D, clamps. Scale bars: A, 500 μm; B, 50 μm; C, 100 μm; D, 20 μm.

*Type-host*: *Diplodus capensis* (Smith) (Sparidae), Cape white seabream.

*Type-locality*: Chintsa East, South Africa.

*Other localities*: Witsand, Mossel Bay, the Tsitsikamma section of the Garden Route National Park, and Chintsa East, South Africa.

*Site of infestation*: gills.

*Deposited examined material*: Holotype (NMBP1125) and 11 paratypes (NMBP1126–NMBP1136), including four hologenophores, are deposited in the NMB; five paratypes deposited in SMNH (Type-9983–Type-9987).

*Representative DNA sequences*: Partial 28S rDNA, five sequences (PV627795–PV627799); Partial COI mtDNA, five sequences (PV612823–PV612827).

*Etymology*: This species is named after the host fish *Diplodus capensis*, for which the commonly used Afrikaans name in South Africa is the dassie (noun in apposition).

#### Description

Body lanceolate (Fig. 5A), occasionally slender, 1892–3510 (2660) long including haptor length; widest at level of ovary 376–652 (512). Haptor spatulate, pointed at anterior and posterior extremities, not well demarcated from body; bearing 64–130 (106) clamps. Clamps of “microcotylid” type, sessile, situated in two parallel rows; clamps decreasing in size towards posterior extremity of haptor; anterior clamps 24–46 (35) long, 44–98 (69) wide; posterior clamps 17–40 (26) long, 28–76 (47) wide. Clamps formed by anterior (Fig. 5B) and posterior jaws (Fig. 5C). Ventral arm of median spring *a1* Y-shaped, long, slender; distal part of *a1* Y-shaped, with pointed short branches of equal size; proximal part *a2* wider, T-shaped, with short branches. Dorsal arm of median spring *a3* shorter than *a1*, distally broad. Ventral arm of ventral jaw sclerites *b1*, dorsal arm *b2* short and curved inwards, *b2* not reaching median spring. Dorsal jaw sclerites *c* shorter than ventral, sclerites *c* not reaching midline on distal side. Muscle connecting *a2* and *b2* present on proximal side (Fig. 5D).

Prohaptoral suckers transversely oval, 79–125 (100) long, 66–97 (84) wide, muscular, divided by transverse muscular partition into two subequal chambers, lateral chamber smaller (Fig. 5E). Pharynx spherical to subspherical, 37–54 (45) long, 34–47 (39) wide. Oesophagus relatively long, with bilateral pair of diverticula. Intestinal bifurcation at level of common genital pore; caeca with numerous lateral and axial ramifications, uniting posteriorly, extends into haptor.

Testes compactly arranged in intercaecal region of middle third of body, obscured by vitellarium. Seminal vesicle opening into male copulatory organ (MCO). MCO pyriform, broader posteriorly, narrowed anteriorly, 28–40 (35) long; MCO consisting of inner tube and outer sheath; inner tube with nearly parallel margins, narrowing slightly before entering distal portion of outer sheath (Fig. 5E). Male accessory gland (MAG) ducts paired, lateral, with proximal bulbous expansion; union of ducts not examined. Genital atrium unarmed, surrounded by a circular layer of concentric muscles. Vas deferens sinuous, wide, dorsal to uterus, extending in intercaecal region anteriorly to MCO; ending in a thick-walled seminal vesicle anteriorly (Fig. 5E).

Germarium pretesticular, intercaecal, question-mark shaped, dorsal to uterus (Fig. 5F); germarium narrowing posteriorly and is followed by oviduct. Oviduct receiving genito-intestinal canal and vitello-vaginal reservoir. Genito-intestinal canal uniting with right intestinal caecum. Oviduct short, ending in an ootype. Mehlis glands not examined. Uterus thin-walled, ascending straight, ending in genital atrium. Vaginal opening ventral, posterior to pore of genital atrium (Fig. 5E). Vagina beginning with thin-walled transversal enlargement; continuing with two parallel thin-walled vaginal ducts. Vaginal ducts opening at junction of transverse vitelloducts. Vitellarium follicular, overlapping intestinal caeca, forming two lateral broad bands, uniting in posterior part of body behind testes, forming a single strip which penetrates axial part of haptor. Efferent vitelline ducts detaching in anterior third of body and uniting on midline; receiving vaginal duct anteriorly and uniting posteriorly forming vitello-vaginal ducts. Lateral vitello-vaginal ducts uniting dorsally, forming vitello-vaginal reservoir. Vitello-vaginal ducts separating into two lateral vitelline ducts posteriorly, uniting again between two transverse parts of ovary, forming common vitelline ducts. Eggs fusiform, with bipolar filaments; anterior filament long, highly coiled distally; posterior filament shorter, digitiform.

#### Remarks

*Polylabris dassie* n. sp. fits the diagnosis of this genus well, by having a sclerotised conical male copulatory organ [60]. Currently, WoRMS [71] lists 23 nominal species of *Polylabris*. However, according to Hayward [26], *P. mamaevi* Ogawa & Egusa, 1980, *P. indica* Hayward, 1996 and *P. virgatarum* (Tubangui, 1931) are *species inquirenda* and are thus not considered herein. WoRMS [71] lists *P. longispinosus* Byrnes, 1985 and *P. mylionis* Dillon, Hargis & Harrises, 1985 as members of *Polylabris* but these species were first described as *Polylabroides longispinosus* Byrnes, 1985 (see [11]) and *Po. mylionis* Dillon, Hargis & Harrises, 1985 [15], and are still maintained as valid species of *Polylabroides* Mamaev & Parukhin, 1976 (see Hayward [26] and the *Addendum* in Mamaev [44]), therefore these species are also not considered herein.

According to the number of vaginae, we followed Hayward [26] who divided the species of *Polylabris* into two groups: the “bivaginate group”, with paired vaginae and the “univaginate group”, with a single vaginal opening. According to Hayward [26] and subsequent descriptions of new species [60, 76], the “bivaginate group” includes six species *P. australiensis* Hayward, 1996; *P. carnarvonensis* Dillon, Hargis & Harrises, 1983; *P. queenslandensis* Hayward, 1996; *P. sigani* Dillon, Hargis & Harrises, 1983; *P. sillaginae* (Woolcock, 1936); and *P. williamsi* Hayward, 1996. The “univaginate group” includes 14 species: *P. acanthogobii* (Yamaguti, 1940); *P. acanthopagri* Mamaev & Parukhin, 1976; *P. angifer* Hussey, 1986; *P. bengalensis* Sailaja & Madhavi, 2011; *P. gerres* (Sandars, 1944); *P. girellae* Hayward, 1996; *P. halichoeres* Wang & Zhang, 1998; *P. japonicus* Ogawa & Egusa, 1980; *P. kuhliae* (Yamaguti, 1968); *P. lingaoensis* Yang, Kritsky & Pan, 2007; *P. madagascariensis*; *P. maomao* (Yamaguti, 1968); *P. rhabdosargi* Hayward, 1996; and *P. tubicirrus* (Paperna & Kohn, 1964).

By having a single mid-ventral vaginal opening, *P*. *dassie* n. sp. is classified within the “univaginate group” and is compared to other members of this group below (see also Supplementary Table S1).

*Polylabris dassie* n. sp. differs from *P. acanthogobii* by having smaller oral suckers (79–125 × 66–97 *vs* 36–54 × 30–48) and a smaller MCO (28–40 *vs* 40–45). The two species can be easily distinguished, as the tip of the MCO of *P. acanthogobii* has a slight constriction before widening and thickening into the dorsal recurvature, and a ventral projection; by possessing male accessory glands that are not constricted; and by having a very stout haptor, with fewer clamps [72]. This new species also differs from *P. acanthopagri* by having a smaller body (1892–3510 × 376–652 *vs* 5030–5800 × 530–560), by having fewer clamps (106 *vs* 160) and a shorter MCO (35 *vs* 50). The two species can be easily distinguished by *P*. *dassie* n. sp. lacking a thin additional process at the posterior end of the main median plate, and by sharp small teeth on the dorsal edge of the MCO. Moreover, *P*. *dassie* n. sp. is readily distinguished from *P. acanthopagri* by lacking the well-marked chamber extensions of vaginal ducts at some distance from the vagina (see Fig. 2b in Mamaev and Parukhin [46]).

The new species differs from *P. angifer* by having fewer clamps (64–130 *vs* 100–140) and a shorter MCO (28–40 *vs* 50–80), by having a straight MCO (*vs* armed with a sharp dorsally curved stylet at its tip in *P. angifer*) and by the vaginal ducts not extending laterally, close to the body margins [29]. Whereas *P*. *dassie* n. sp. can also be distinguished from *P. bengalensis* by having more clamps [64–130 (106) *vs* 64–78 (68)] and a smaller MCO [28–40 (35) *vs* 44–60 (54). Additionally, *Polylabris dassie* n. sp. is easily distinguished by having confluent vitelline fields and caeca that unite posteriorly (*vs* caeca ending blindly in *P. bengalensis*) [60].

*Polylabris dassie* n. sp. differs from *P. gerres* by having larger clamps (35 × 69 *vs* 25 × 58), larger oral suckers (100 × 84 *vs* 63) and a smaller MCO (35 *vs* 46) [61]. The new species is easily distinguished by having a straight MCO (*vs* anteriorly curved stylet at its tip in *P. gerres*), and by lacking the well-marked chamber extensions of the vaginal ducts (see Figure 3b in Mamaev and Parukhin [46]). Additionally, this new species differs from *P. girellae* by having more (64–130 (106) *vs* 66–78 (74)) and smaller (24–46 (35) × 44–98 (69) *vs* 42–51 (48) × 77–94 (88)) clamps, smaller oral suckers (79–125 (100) × 66–97 (84) *vs* 75–98 (86) × 24–71 (49)) and a smaller MCO (28–40 (35) *vs* 54–61 (58)) [26]. *Polylabris dassie* n. sp. can also be differentiated from *P*. *halichoeres* by having far more clamps on the haptor (64–130 *vs* 56–64), as well as notably larger oral suckers (79–125 × 66–97 *vs* 55–61 × 40–43) [79].

The new species differs from *P. japonicus* by having a smaller body (1892–3510 × 376–652 *vs* 3800–5000 × 830–930), smaller clamps (24–46 × 44–98 *vs* 77–91), and smaller MCO (28–40 *vs* 47–54). *Polylabris dassie* n. sp. can easily be distinguished from this species by the caeca uniting posteriorly (*vs* ending blindly in *P. japonicus*), and by lacking the chamber-like extensions of the vaginal ducts [52]. *Polylabris dassie* n. sp. can also be distinguished from *P. kuhliae* by having larger oral suckers (79–125 × 66–97 *vs* 37–63 × 45–63) and a smaller MCO (28–40 *vs* 42–50). The two species are readily distinguished by the new species lacking a styliform piece on the apex of the median spring of the clamps, and the caeca uniting posteriorly (*vs* ending blindly in *P. kuhliae*) [74].

*Polylabris dassie* n. sp. differs from *P. lingaoensis* by having a larger body (1892–3510 (2660) × 376–652 (512) *vs* 1130–1597 (1356) × 159–298 (236)), more numerous (64–130 *vs* 60–86) and larger clamps (33–40 (36) *vs* 44–98 (69)). Additionally, *Polylabris dassie* n. sp. can be easily distinguished from *P. lingaoensis* by possessing an MCO with a straight tip (*vs* dorsally recurved in *P. lingaoensis*) [76].

*Polylabris dassie* n. sp. differs from *P. madagascariensis* by having a larger body (1892–3510 × 376–652 *vs* 1350–1810 × 225–270), larger clamps (24–46 × 44–98 *vs* 32–34 × 59–63), double the number of clamps (64–130 *vs* 60–62) and a smaller MCO (28–40 *vs* 40–43). The two species can be easily distinguished by the tip of the MCO being strongly recurved dorsally in *P. madagascariensis*. Despite both species being reported from South Africa, the localities are distinct, as *P. madagascariensis* was first described from the sub-tropical East Coast of South Africa (Sodwana Bay) while *P*. *dassie* n. sp. is reported from the temperate South Coast of South Africa. Additionally, the host families of these two species are distinct (Sparidae *vs* Sillaginidae) [26].

*Polylabris maomao* can be distinguished from the new species by having a recurved, tapering tip of the MCO [74], whereas *P. dassie* n. sp. differs from *P. rhabdosargi* by having smaller clamps (24–46 (35) × 44–98 (69) *vs* 41–50 (45) × 80–91 (86)) and a smaller MCO (28–40 (35) × 41–51 (45)) [26]. Lastly, *P*. *dassie* n. sp. differs from *P*. *tubicirrus* by possessing a shorter body, slightly less clamps, larger oral suckers and a shorter MCO [54].

### Molecular characterisation

#### 28S rDNA

**Figure 7:**
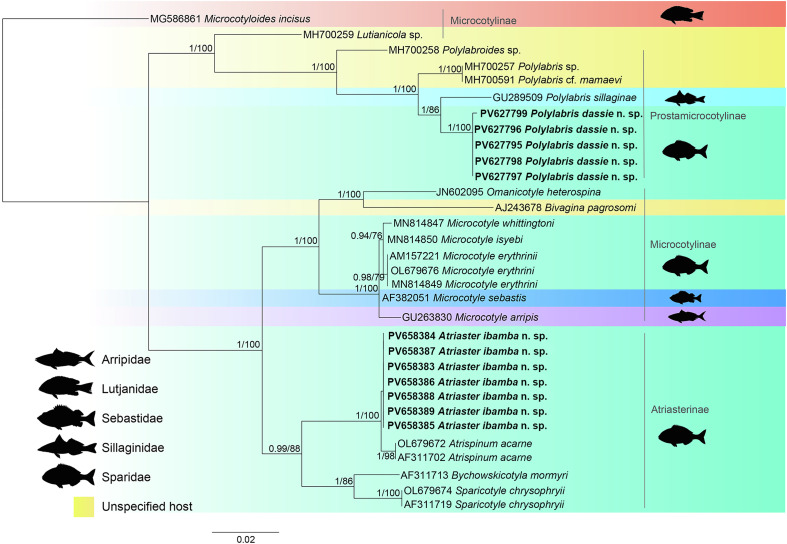
Maximum likelihood phylogram based on partial sequences of the 28S rDNA gene of selected polyopisthocotylan species. Newly sequenced isolates are in bold. Posterior probability followed by bootstrap support values are given next to the branches (posterior probability < 0.90 and bootstrap < 60 not shown). *Microcotyloides incisus* (Linton, 1910) was used as an outgroup. Branch length scale bar indicates number of substitutions per site.

Partial 28S rDNA sequences were generated for seven isolates of *A. ibamba* n. sp. (1297–1580 bp long), and for five isolates of *P. dassie* n. sp. (1470–1591 bp long), parasitising *D. capensis* collected off the South Coast of South Africa. The newly generated sequences were compared to selected sequences of Atriasterinae, Microcotylinae and Prostatomicrocotylinae available in GenBank (Table 2; Fig. 7). *Microcotyloides incisus* (Linton, 1910) (MG586861) [49] was used as an outgroup. The trimmed matrix included 843 base pairs.

The newly generated sequences of *A. ibamba* n. sp. clustered together in a strongly supported clade, and as a sister group to *Atrispinum acarne* Maillard & Noisy, 1979 (OL679672 and AF311702). A larger strongly supported clade was formed with isolates of *Bychowskicotyla mormyri* (Lorenz, 1878) (AF311713) and *Sparicotyle chrysophrii* (Van Beneden & Hesse, 1863) (OL679674 and AF311719), which are all part of the Atriasterinae. Figure 7 shows the phylogenetic relationships of the isolates included in the analyses of the partial 28S rDNA. The newly generated isolates of *P. dassie* n. sp. clustered together and as a sister group to one isolate of *P. sillaginae* (GU289509). A strongly supported larger clade was formed including the aforementioned isolates, one isolate of *Polylabroides* sp. (MH700258), one isolate of *Polylabris* sp. (MH700257) and *Polylabris* cf. *mamaevi* (MH700591), which are all part of the Prostamicrocotylinae. The species of the Microcotylinae, other than the one used as an outgroup, clustered together in a strongly supported clade.

The newly sequenced isolates of *A. ibamba* n. sp. are identical. They differ from *At*. *acarne* isolates by only 0.4–0.6% (3–4 bp). Newly sequenced isolates of *P. dassie* n. sp. are identical, except for isolate 18, which differed from all the other isolates by 0.1% (1 bp), which can be considered intraspecific. They differed from *P. sillaginae* by 2.3–2.4% (19–20 bp), and from *Polylabris* sp. and *P*. cf. *mamaevi*, which are identical sequences, by 2.4–2.8% (20–23 bp). Supplementary data show the values of genetic distances among all sequences included in the 28S rDNA phylogenetic analyses (Table S2).

#### COI mtDNAs

**Figure 8:**
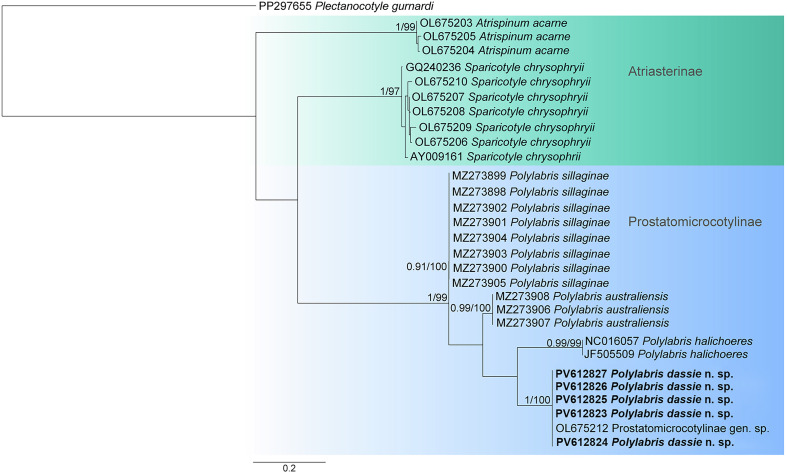
Maximum likelihood phylogram based on sequences of the COI mtDNA gene of selected polyopisthocotylan species. Newly sequenced isolates are in bold. Posterior probability followed by bootstrap support values are given next to the branches (posterior probability < 0.90 and bootstrap < 60 not shown). *Plectanocotyle gurnardi* (Van Beneden & Hesse, 1863) Llewellyn, 1941 was used as an outgroup. Branch length scale bar indicates number of substitutions per site.

Partial COI mtDNA sequences were generated for five isolates of *P. dassie* n. sp. retrieved from *D. capensis* from four localities along the South African South Coast. The newly generated *Polylabris* COI sequences were analysed together with sequences of the Prostatomicrocotylinae and Atriasterinae available in GenBank (Table 2; Fig. 8). The plectanocotylid, *Plectanocotyle gurnardi* (Van Beneden & Hesse, 1863) (PP297655) [12] was selected as an outgroup. The trimmed matrix included 281 positions. The genetic code Flatworm Mitochondrial (Frame 2) was applied for translation into amino acids.

The five isolates of *P. dassie* n. sp. clustered together in a well-supported clade, supporting the presence of a single species, *P*. *dassie* n. sp. The isolate Prostatomicrocotylinae gen. sp. (OL675212) nested within the *P. dassie* n. sp. clade, suggesting that it might represent the new species of *Polylabris* described herein. The COI tree showed the clade composed of *At*. *acarne* isolates (OL675203–OL675205) as a basal clade. A larger clade split into two lineages: one encompassing isolates of *S. chrysophrii*, and another encompassing *Polylabris* spp.

The newly generated sequences of *P. dassie* n. sp. are identical, as well as the sequence OL675212, suggesting their conspecificity. The isolates of *P. dassie* n. sp. differed by 14.6% (41 bp) from those of *P. halichoeres*, by 12.1% (34 bp) from *P. australiensis*, and by 14.6% (41 bp) from *P. sillaginae*. Supplementary data shows the values of genetic distances amongst all sequences included in the COI mtDNA phylogenetic analyses (Table S2).

## Discussion

This is the first report of a polyopisthocotylan species from *D*. *capensis* in South Africa. The lack of knowledge about this parasitic taxon from a previously studied and widely favoured angling fish may be attributed to the scarcity of taxonomists that focus on the polyopisthocotylans of marine teleost fishes within this geographic region.

Through an integrative taxonomic approach, two species of the class Polyopisthocotyla collected from *D*. *capensis* were identified as new to science: *A. ibamba* n. sp. and *P. dassie* n. sp. This is the first report of a species of *Atriaster* from South Africa and we provide the first molecular sequences based on the partial 28S rDNA for microcotylids of this genus. The most recent and complete phylogeny of the Microcotylidae is that of Lablack *et al.* [32], in which the Atriasterinae available in GenBank were represented by *Sparicotyle* Mamaev, 1984, *Bychowskicotyla* Unnithan, 1971, and *Atrispinum* Euzet & Maillard, 1974. Herein, we add sequences (accompanied by permanently mounted and illustrated hologenophores) of a fourth genus, *Atriaster*. Despite the lack of available sequences for species of *Atriaster* in GenBank, the newly collected species described here as *A*. *ibamba* n. sp., corresponds well to the generic diagnosis of *Atriaster* by possessing a pair of longer spines at the top of the crown of spines that surround the genital atrium, two vaginae and a supporting ribbed plate in the genital atrium [35].

Currently, *Atriaster* includes five valid species [70], of which three were first described from the Indian Ocean (*A. acanthopagri*, *A. bifidacanthus* and *A. spinifer*) while the remaining were first described from the Atlantic (*A. heterodus* and *A. maillardi*). The novel species is described from the South Coast of South Africa, where the Indian and Atlantic oceans meet to create unique conditions that drive biodiversity.

Interestingly, all species of *Atriaster* are hosted by sparid fishes: *A. bifidacanthus* parasitises *Sparus* sp. [45], *A. maillardi* parasitises the zebra seabream *Diplodus cervinus*, *A*. *heterodus* was described from a sparid fish host [35], *A. acanthopagri* and *A. spinifer* both parasitise the twobar seabream *Acanthopagrus bifasciatus* [45], and *A. spinifer* was described from the king soldierbream *Argyrops spinifer* and *A*. *bifasciatus* [45]. Hence, *Atriaster* exhibits a stenoxenic specificity for sparid fishes. This is not unusual for Atriasterinae, for instance, all *Atrispinum* spp. were first described from sparid hosts: *At*. *acarne* was first described from the axillary seabream *Pagellus acarne* [42], *Atrispinum salpae* (Parona & Perugia, 1890) was first described from the Salema *Sarpa salpa*, and both *Atrispinum sargi* (Parona & Perugia, 1890) and *Atrispinum seminalis* (Euzet & Maillard, 1973) were both first described from the white seabream *D. sargus*, the common two-banded seabream *D. vulgaris*, and the annular seabream *D. annularis* [19].

A similar host specificity pattern has been examined in several polyopisthocotylans. For instance, most *Gastrocotyle* spp. (Gastrocotylidae) parasitise mainly Carangidae [3, 34, 64, 66]; *Intracotyle* spp. (Microcotylidae) are mainly known from Haemulidae [17, 33, 43, 65, 68]; *Cemocotyle* spp. (Heteraxinidae) are known only from Carangidae [14, 40, 47, 56]; *Cotyloatlantica* spp. (Chauhaneidae) and *Rhinecotyle* spp. (Rhinecotylidae) are known only from *Sphyraena* spp. [8, 20]; and *Pyragraphorus* spp. (Pyragraphoridae) are known only from Carangidae [18, 41, 63, 73, 78].

*Polylabris* is the most speciose of the Prostatomicrocotylinae and differs from other genera by its species having a sclerotised male copulatory organ. Interestingly, the newly generated COI sequences of *P*. *dassie* n. sp. are identical to those of a Prostatomicrocotylinae sp. found from *D. vulgaris* off Algeria, Western Mediterranean [32]. Overall, the widespread distribution of polyopisthocotylans across different regions is not uncommon and the occurrence of a single species in distinct localities has been previously verified using COI barcodes. Cappelletti and Bouguerche [12] summarised cases where the taxonomic and geographic status of several polyopisthocotylans in different localities has been validated with COI barcodes. For instance, *Allogastrocotyle bivaginalis* Nasir & Fuentes Zambrano, 1984 (sensu Bouguerche *et al.* [5]) occurs both in Mediterranean waters off Algeria [5] and in Australian waters off the southwest Pacific [28]; *Kuhnia scombri* (Kuhn, 1829) and *Pseudokuhnia minor* (Goto, 1984) were demonstrated to occur in ten localities along the coast of China [75] as well as off Australia [28]. Thus, considering the available data, the species of Prostatomicrocotylinae reported by Lablack *et al.* [32] is very likely conspecific with *P*. *dassie* n. sp., but morphological comparison with voucher specimens is needed to provide a definitive conclusion.

## Conclusion

*Atriaster ibamba* n. sp. and *P*. *dassie* n. sp. are the first members of the Microcotylidae to be genetically characterised from the South Coast of South Africa, which will contribute to a better understanding of the phylogeography and delineation of this family in future studies. Considering the unique coastal habitats of South Africa, which is known to be one of the most biodiverse countries in the world, a greater diversity of these parasites would be expected. Therefore, conducting more explorative taxonomic studies on these marine parasites in this region will be highly beneficial for future work on this group.

## Supplementary materials

The supplementary material for this article can be found at https://www.parasite-journal.org/10.1051/parasite/2025037/olm. *Table S1*: Morphometric data for all valid species of *Atriaster* and *Polylabris*, including that of the new species described herein. *Table S2*: Genetic divergences between species included in the phylogenetic analyses.
